# Organoid models: applications and research advances in gastric cancer

**DOI:** 10.3389/fonc.2026.1766571

**Published:** 2026-04-17

**Authors:** Rui Ling, Yuning Wu, Zhiying Yan, Qingying Xian, Yunqian Chu, Wenyu Zhu

**Affiliations:** Department of Oncology, The Third Affiliated Hospital of Nanjing Medical University, Changzhou Second People’s Hospital, Changzhou, China

**Keywords:** organoids, gastric cancer, *helicobacter pylori*, drug screening, individualized treatment, applications

## Abstract

Gastric cancer research necessitates representative experimental models due to its profound heterogeneity. This review summarizes the establishment of human gastric organoids derived from adult and pluripotent stem cells and categorizes five representative culture models, including recently developed bioengineered platforms. We synthesize recent applications of gastric organoid systems across major research contexts in gastric cancer and discuss their extension toward more complex co-culture configurations and therapy-associated tumor evolution models. Finally, this review outlines current technical, translational, and ethical challenges to provide an integrated perspective on the future development of gastric organoid-based research.

## 1 Introduction

Gastric cancer (GC) is the fifth most common cancer worldwide and the third leading cause of cancer-related death, with more than one million new cases diagnosed annually (1). Despite advances in multidisciplinary treatment strategies, including perioperative S-1 plus oxaliplatin (SOX) regimen and immunotherapy-based combinations, long-term outcomes for patients with advanced GC remain suboptimal (2, 3). A major challenge is the pronounced molecular and genetic heterogeneity of GC, which limits the effectiveness of standardized treatment strategies in precision oncology (4).

In routine clinical practice, imaging and endoscopic biopsies provide limited information and cannot fully capture spatial tumor heterogeneity or real-time tumor evolution during treatment (5). Furthermore, therapeutic efficacy is frequently constrained by the absence of reliable predictive tools for defining individual drug responses. Even patients with identical clinical stages often exhibit vastly different sensitivities to the same regimen, leading to primary resistance or unnecessary systemic toxicity (6). These limitations highlight the need for experimental platforms that better connect laboratory research with clinical applications and support individualized therapeutic decision-making.

Conventional *in vitro* and *in vivo* models face inherent constraints in balancing physiological relevance with the logistical demands of time-sensitive clinical applications (7, 8). Against this background, patient-derived organoids (PDOs) have emerged as an experimental platform for modeling GC (9–14).

**Figure 1:**
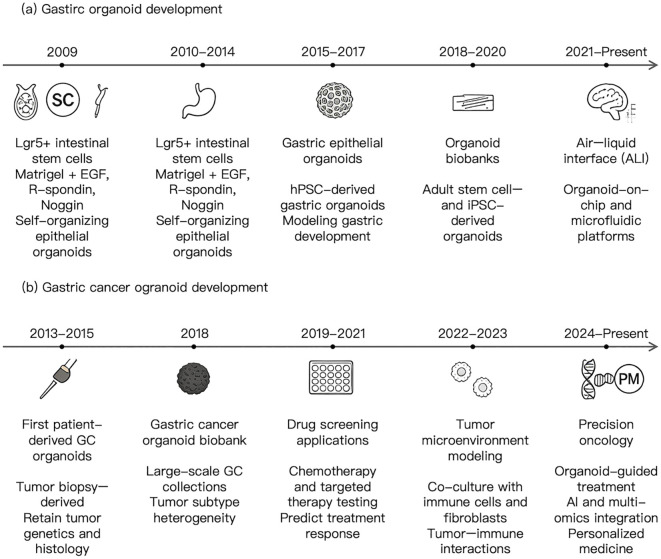
Developmental timeline of gastric and gastric cancer organoids. **(a)** Schematic representation of the key milestones in the establishment and evolution of gastric organoid systems, from early *in vitro* culture techniques to the generation of long-term, self-organizing three-dimensional gastric epithelial structures. This panel highlights major advances in stem cell (SC)-derived and adult tissue–derived organoid models, including improvements in culture conditions, niche factor optimization, and the ability to recapitulate physiological architecture and cellular heterogeneity of the gastric epithelium. **(b)** Timeline illustrating the development of gastric cancer (GC) organoids, emphasizing the transition from conventional cancer models to patient-derived organoid platforms. Key milestones include the successful establishment of tumor organoids from primary gastric cancer tissues, the preservation of histopathological and genetic characteristics, and the application of these models in functional studies. The panel further summarizes the integration of gastric cancer organoids into translational research, including drug screening, biomarker discovery, tumor heterogeneity analysis, and precision oncology and personalized medicine (PM). Representative studies and major technological advances are indicated along the timeline to provide a comprehensive overview of the field.

This review summarizes the progress of GC organoid research through a three-tier conceptual framework: (a) mechanistic modeling for dissecting signaling and resistance; (b) translational prediction for individualized therapeutic anticipation; and (c) systems-level integration for modeling complex microenvironment interactions and multi-omics evolution (**Figure 1**).

## 2 The evolution of organoids

Gastric organoid technology has evolved through several advances designed to address the limitations of earlier experimental models. Initial efforts were constrained by the lack of stable human gastric epithelial cultures. This limitation was addressed through the identification of leucine-rich repeat–containing G protein-coupled receptor 5–positive (LGR5^+^) gastric stem cells and the establishment of Matrigel-based niche cultures supplemented with epidermal growth factor (EGF), R-spondin, and noggin, enabling long-term expansion of gastric epithelium and the formation of gland-like structures *in vitro* (15–17).

Despite these advances, the limited availability of primary human gastric tissue restricted large-scale experimental applications. The development of induced pluripotent stem cell (iPSC)-derived gastric organoids provided a renewable human model for studying gastric development and disease processes, including host–pathogen interactions such as *Helicobacter pylori* infection (12, 18, 19).

As research priorities shifted toward functional modeling, conventional submerged cultures showed incomplete epithelial maturation and limited mucus production. The adoption of air–liquid interface (ALI) systems improved epithelial polarization and mucous cell differentiation, thereby enhancing the physiological relevance of gastric mucosal barrier models and infection studies (20, 21).

Static culture conditions do not capture gastric-specific biomechanical signals such as luminal shear stress and peristaltic-like strain, which limits the predictive value of organoids for drug testing. Microfluidic stomach-on-a-chip platforms address these issues by introducing controlled perfusion and microenvironmental regulation. These systems enable the dynamic simulation of gastric fluid flow and drug transport through the mucus barrier, thereby providing a more physiologically relevant platform for studying pharmacokinetics and epithelial barrier function in gastric models (22–25).

**Figure 2:**
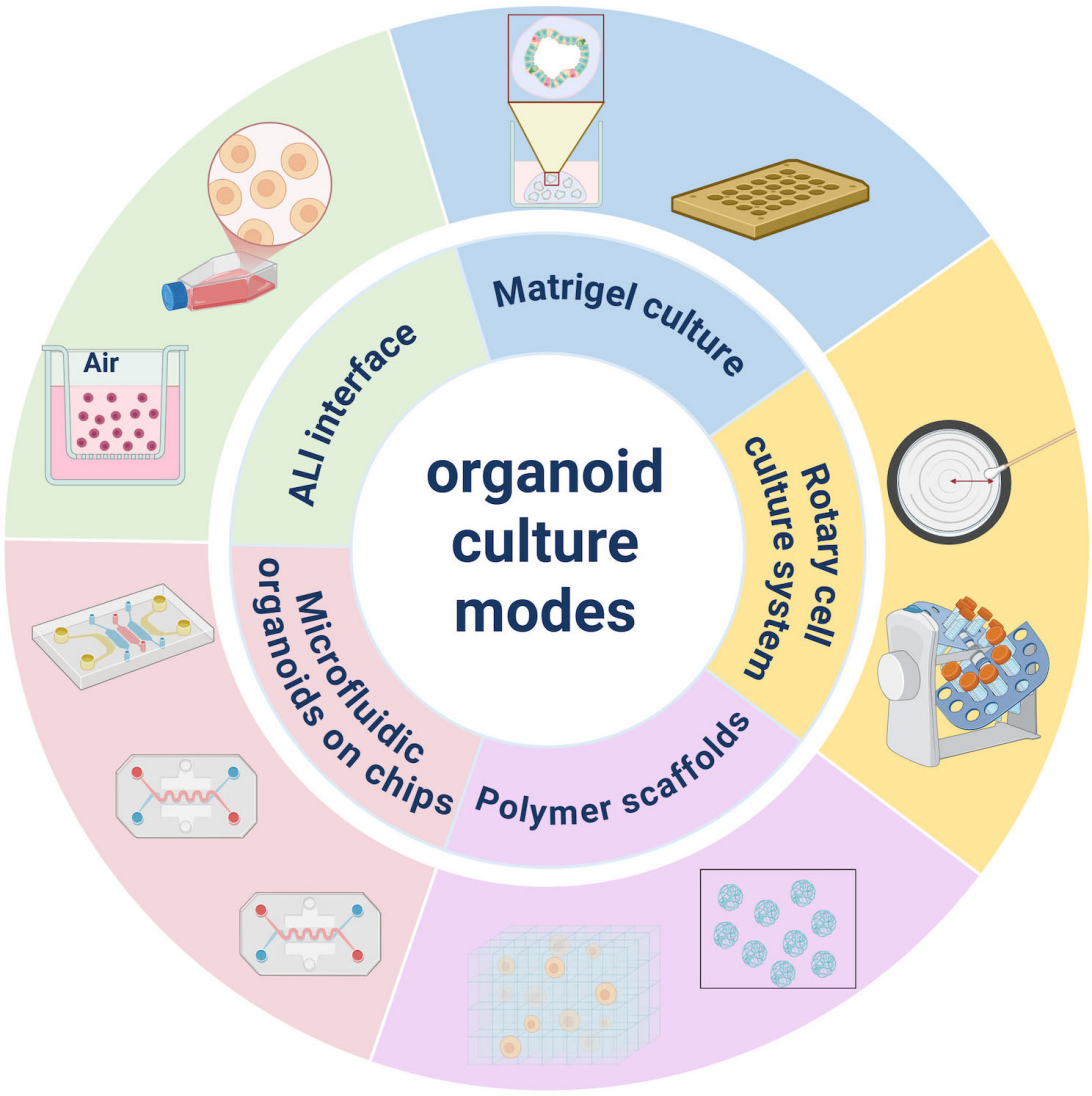
Culture modes of patient-derived organoids in gastric cancer. Culture modes of patient-derived organoids in gastric cancer: Matrigel culture, Rotary cell culture system, Air-liquid interface, Polymer scaffolds, and Microfluidic organoids on chips. The selection of a specific culture mode is driven by the distinct biological questions and clinical requirements of gastric cancer research. Matrigel culture is the most classical and cost-effective modality, widely used to establish patient-derived organoid biobanks and to perform high-throughput drug screening to evaluate chemoresistance. Polymer scaffolds utilize 3D bioprinting to artificially construct the extracellular matrix, allowing researchers to adjust the spatial positioning of cells and study the impact of structural cues on tumor growth. The Rotary Cell Culture System creates a microgravity-like suspension that minimizes shear stress and internal necrosis, making it ideal for the long-term expansion of large-scale gastric organoids with high cellular viability. The air-liquid interface provides a high-oxygen environment and facilitates the formation of a robust mucus layer, which is essential for modeling *Helicobacter pylor****i*** infections and studying immune-epithelial interactions within the gastric niche. Finally, Microfluidic organoids on chips integrate micro-channels to simulate dynamic fluid-tissue interactions and gastric peristalsis, providing a high-fidelity platform for pharmacokinetic modeling and personalized oncology. Researchers should prioritize Rotary cell culture system or Air-liquid interface for complex physiological modeling, whereas Matrigel-based models remain the standard for initial clinical diagnostic screening due to their scalability and reproducibility.

Furthermore, for patients with advanced GC where biopsy material is scarce, these platforms increase the efficiency of PDO establishment by optimizing nutrient delivery in confined volumes (26, 27). Consequently, advances in gastric organoid technology have provided a useful platform for disease modeling (**Figure 2**).

By preserving key genetic features and tissue architecture of the human stomach, such systems provide functional advantages over conventional systems. These advances provide a basis for translating mechanistic insights into potential therapeutic strategies for GC.

## 3 Advantages over traditional models

To evaluate the advantages of PDOs in cancer research, their performance can be compared with two-dimensional (2D) cell culture and patient-derived xenograft (PDX) models (**Table 1**). 2D cell culture was the earliest *in vitro* model used for GC research. This model offers advantages including ease of operation, low cost, and good reproducibility, and is widely used in basic research and drug screening (28, 29). However, prolonged monolayer growth under non-physiological conditions makes cells prone to genetic and phenotypic drift. As a result, these models cannot fully recapitulate the complex features of native tumors (30, 31). In contrast to conventional 2D cell lines and *in vivo* models, organoid technology provides tumor models that more closely resemble the physiological characteristics of native tumors. They encompass diverse cell lines and spatial organization, closely mimicking the genomic and cellular diversity inherent to primary cancer. Consequently, these models are useful for disease modeling, investigation of resistance mechanisms, and patient-specific drug testing (32). The primary limitations of conventional models include high construction costs, stringent cultivation dependencies, and a lack of standardization. PDX models offer more physiologically relevant information, but their application is constrained by factors such as high costs, lengthy modeling cycles, and interspecies differences (33–35). PDOs preserve the morphological and genomic features of the original tumors and typically expand within 4–6 weeks (7, 29, 36). This approach accelerates preclinical drug validation and supports precision oncology by helping identify effective treatment regimens and potentially reducing unnecessary toxicity (8, 37, 38).

**Table 1:** Comparison between 2-dimensional cell culture, patient-derived organoid, and patient-derived tumor xenograft models.

Feature	2D cell culture	PDO	PDX
Cost	Low	Moderate	High
Ease of maintenance	Easy	Moderate	Difficult
Time requirement	Short	Moderate (4–6 weeks)	Long
Success rate of establishment	High (~100% for established lines)	Moderate (50–76% in GC; variable by tumor type)	Moderate (50–60%)
Long-term stability	Limited	Moderate	High
Heterogeneity	Low	Moderate	High
High-throughput drug screening	High	Moderate	Low
Host-microbe interaction studies	Moderate	Limited (enhanced with ALI/co-culture)	Limited
Immune evaluation	Not suitable	Limited	Moderate (systemic immune components)
Drug response prediction	Low	Moderate (biased subset; context-dependent)	Moderate (debated; systemic responses)
Tumor microenvironment modeling	Low	Moderate (missing vasculature/immune/stroma)	High
Physiological relevance	Low	Moderate	High
Disease modeling fidelity	Low	Moderate	High
Reproducibility	High	Moderate	Low

2D, 2-dimensional; PDO, Patient-derived organoid; PDX, Patient-derived tumor xenograft; ALI, Air-Liquid Interface; GC, Gastric Cancer.

Despite their high-throughput potential, PDO models still have several limitations. These include dependence on specific niche factors and the absence of a complete tumor microenvironment (TME), particularly stromal components, functional vasculature, and immune cells (32). PDX models uniquely capture systemic tumor–host interactions within an intact organism, including native endocrine systems and neuro-hormonal regulation that organoids cannot yet replicate. This integrated microenvironment is crucial for studying complex processes like spontaneous metastasis, organ-specific tropism, and long-term systemic pharmacokinetics/pharmacodynamics. Consequently, researchers employ a strategic workflow: utilizing PDOs for initial candidate filtering and reserving PDX for final high-fidelity validation of systemic drug efficacy and toxicity. This complementary strategy helps ensure that preclinical findings are evaluated in models that better represent systemic tumor–host interactions (39).

## 4 Methodological framework for patient-derived organoid construction

### 4.1 Divergent strategies: pluripotent and adult stem cell-derived models

Translating the inherent physiological and genetic advantages of PDOs into reliable research tools requires precise and reproducible construction strategies (40, 41). PDOs are mainly categorized into two types: those derived from adult stem cells (ASCs) and those derived from pluripotent stem cells (PSCs), which include embryonic stem cells and iPSCs (42, 43). ASCs are typically isolated from glandular tissues or tissue-resident stem cell populations. These cells exhibit multipotent self-renewal capacity and play important roles in tissue repair and regeneration *in vivo*. Importantly, ASC-derived organoids retain regional characteristics of the original tissue and display relatively stable phenotypes, making them suitable for disease modeling and personalized therapy research (44).

Organoids derived from iPSCs, however, undergo germ layer induction and multistage differentiation, enabling them to mimic organ development processes and making them suitable for research on developmental biology (45–48). The generation of iPSC-derived gastric cells begins with the induction of definitive endoderm by treating human pluripotent stem cells with Activin A. Subsequently, bone morphogenetic protein (BMP) inhibitors, fibroblast growth factor (FGF), and Wnt activators are applied to direct cells toward a foregut fate. Retinoic acid is then introduced to further specify gastric lineage differentiation while maintaining high expression of the key transcription factor SRY-box transcription factor 2, an important regulator of gastric epithelial identity (16).

To construct structures with characteristics of the gastric fundic gland, it is also necessary to activate the Wnt/β-catenin signaling pathway at specific developmental stages and simultaneously apply EGF and FGF10 to support the maturation of vesicles. During the late differentiation stage, controlled EGF levels combined with BMP4 and mitogen-activated protein kinase kinase inhibitors promote differentiation into functional gastric cell types, including endocrine and parietal cells. Finally, under high-concentration EGF stimulation, these cells self-organize into human gastric organoids. This developmental process mimics the molecular and morphogenetic stages of gastric antrum formation (49, 50).

ASC-based strategies typically involve isolating intact gastric glands through enzymatic digestion or further dissociating them into individual gastric stem cells (51). Using an improved mini-intestine culture system, single cells are cultured into long-term amplified gastric organoids that highly mimic mature pyloric epithelium. These organoids can be sampled from multiple clinical sources, such as ascites samples, endoscopic biopsy specimens, and surgical resection specimens (52–54). After tissue samples undergo mincing, enzymatic digestion, or mechanical processing, they are similarly embedded in Matrigel for 3D culture. The medium formulation is adjusted according to tumor type and growth characteristics. Compared with normal gastric organoids, which require complete medium for sustained long-term proliferation, cancer organoids often show reduced dependence on certain growth factors. For instance, removing A83-01 (a selective transforming growth factor-beta/activin-like kinase 5 [TGF-β/ALK5] receptor inhibitor), FGF10, or Wnt does not significantly affect GC organoid morphology or growth capacity, suggesting they have gained autonomy in certain signaling pathways (55–57). Furthermore, different factors exhibit varying degrees of maintenance efficacy for cancer organoids, with differing importance attributed to noggin, EGF, and Wnt/R-spondin, reflecting altered microenvironmental dependency in tumor cells.

The key distinction between these approaches lies in their biological basis. The iPSC-based strategy follows principles of developmental biology, progressively guiding pluripotent stem cells toward gastric lineage differentiation and partially recapitulating stomach development. In contrast, the ASCs strategy utilizes stem cells or dedifferentiated cells inherent in adult tissues to directly expand gastric organoids under culture conditions that simulate the stem cell microenvironment (58). This approach is more suitable for modeling adult gastric diseases and regenerative medicine applications.

**Figure 3:**
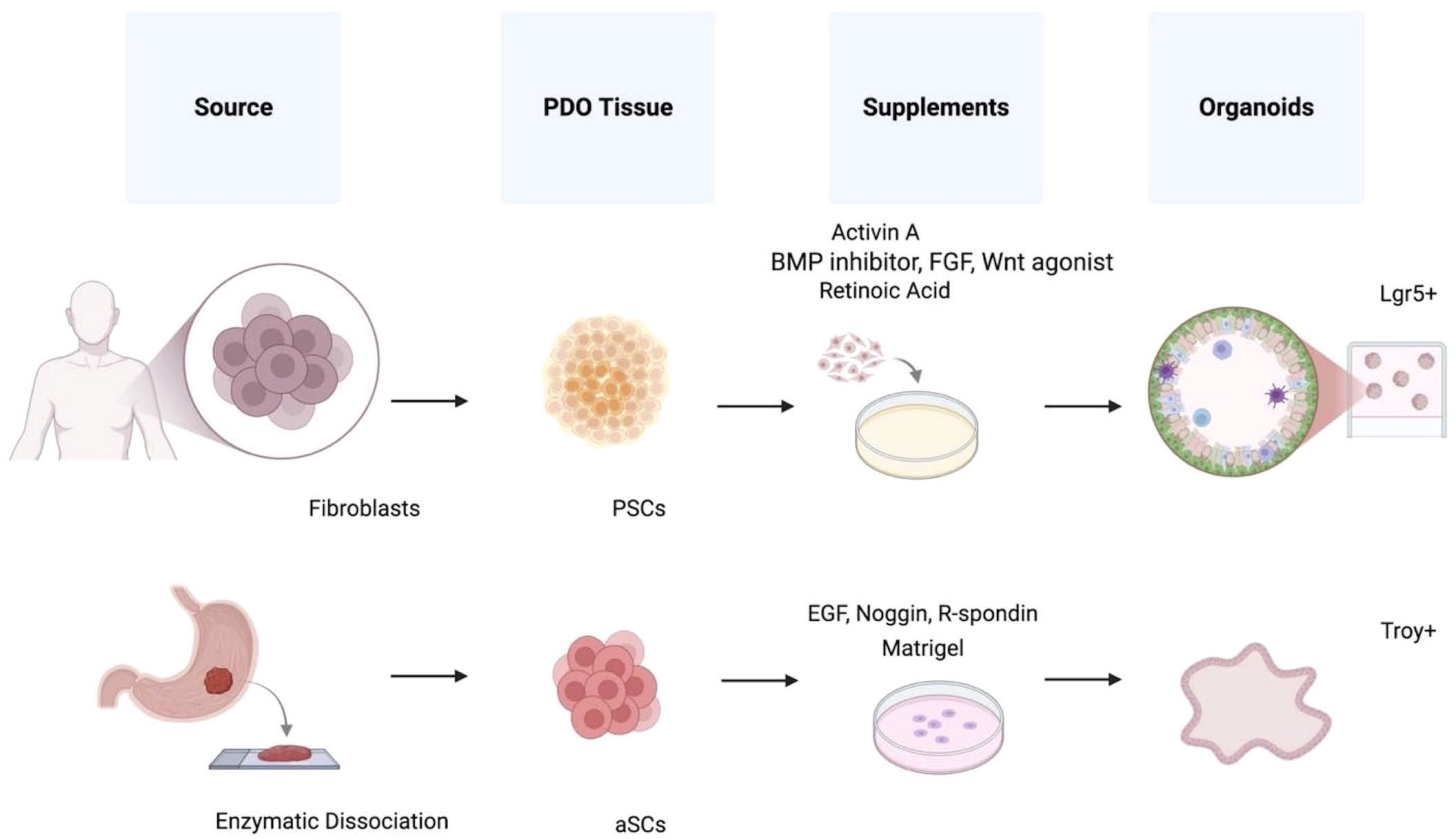
Gastric organoids from different stem cells. Gastric organoids are generated via two distinct pathways, each characterized by specific technical trade-offs and biological advantages for research and clinical translation. The pluripotent stem cell (PSC) strategy involves multi-stage induction: Induced PSCs (iPSCs) are treated with activin A for endoderm induction, followed by bone morphogenetic protein (BMP) inhibitors, fibroblast growth factor (FGF), Wnt agonists, and retinoic acid to mimic embryonic development. Although this pathway requires a significant modeling time frame of 1 to 2 months, it offers superior biological complexity by differentiating into epithelial cells and various stromal cells (e.g., smooth muscle cells and fibroblasts). Consequently, the PSC strategy is the preferred model for developmental biology and studies of the complex gastric niche. In contrast, the adult stem cell (ASC) strategy relies on the direct expansion of tissue-resident stem cells. These can be isolated via invasive methods, such as endoscopic biopsies or surgical resections, which provide high structural similarity to adult tissue, or through less invasive liquid biopsies (e.g., ascites or circulating tumor cells) that allow for dynamic monitoring of disease progression. The ASC pathway is highly efficient, typically requiring only 1 to 2 weeks to obtain stable models. Although limited to epithelial-derived cell populations (e.g., principal and parietal cells), ASC-derived patient-derived organoids (PDOs) exhibit greater proliferative capacity and genetic stability, making them the gold standard for high-throughput drug screening and personalized oncology. After enzymatic dissociation, cells from both pathways are embedded in Matrigel and supplemented with essential niche factors, epidermal growth factor (EGF), noggin, and R-spondin, to form mature organoids expressing markers such as leucine-rich repeat-containing G protein-coupled receptor 5 (Lgr5) and tumor necrosis factor receptor superfamily member 19. By intersecting these technical choices with clinical needs, researchers can select the optimal model (i.e., PSC-derived organoids for mechanistic studies of tissue morphogenesis, or ASC-derived PDOs to restore tumor heterogeneity) and identify patient-specific therapeutic targets.

In summary, standardized isolation and 3D expansion of gastric stem cells have established a robust foundation for generating high-fidelity gastric organoid models. Further optimization of organoid construction strategies will facilitate the development of functional assays and support future clinical translation (**Figure 3**).

### 4.2 Technical determinants of biological interpretation

While tissue source and culture format are often described as methodological variables, they fundamentally determine the biological interpretation of experimental outcomes. Different establishment strategies influence lineage composition, genetic stability, and clonal representation, thereby shaping conclusions regarding tumor heterogeneity and therapeutic sensitivity.

Similarly, culture formats are not merely engineering refinements but biological filters that modulate epithelial maturation, barrier function, metabolic gradients, and immune interactions. For example, submerged systems may favor proliferative signaling readouts, whereas ALI and microfluidic platforms more accurately recapitulate mucosal defense, biomechanical stress, and drug absorption dynamics.

Therefore, GC organoids should not be viewed as a single uniform model system. Instead, they represent a spectrum of technically defined biological contexts. Careful alignment between experimental questions and platform configuration is essential to avoid overinterpretation and to ensure translational validity. This conceptual distinction provides a critical bridge between organoid construction and their downstream applications in mechanistic studies, drug screening, and ecosystem-level modeling.

## 5 Application of organoids in gastric cancer treatment

The application of PDOs has evolved from simple phenotypic observation to a structured hierarchy of research methodologies. In terms of translational prediction, PDOs act as functional diagnostic surrogates, moving beyond static genomic profiling to actively anticipate patient-specific responses to chemotherapy and targeted agents. Organoids can also be integrated into bioengineered systems to model complex TME interactions and multi-omics-driven disease evolution. This hierarchical perspective allows for a more rigorous evaluation of the strengths and limitations inherent in each organoid-based approach.

### 5.1 Mechanistic modeling: dissecting pathogenesis and resistance pathways

#### 5.1.1 Application of gastric cancer organoids in *H. pylori* research

As a major etiological factor in gastric carcinogenesis, *Helicobacter pylori* infection represents an important context in which organoid models provide mechanistic insights. Gastric cancer organoids enable controlled investigation of host–pathogen interactions and their role in tumorigenesis (59). Mechanistically, the bacterial oncoprotein CagA can be stabilized through interactions with host proteins such as AU-rich element RNA-binding protein 1, which inhibits lysosomal degradation and promotes intracellular accumulation of CagA. This process facilitates exosome-mediated dissemination and enhances inflammatory signaling associated with malignant progression (60, 61). Organoid infection models have also demonstrated that inhibition of epidermal growth factor receptor (EGFR) or protein kinase B (AKT) signaling disrupts the interaction between *CagA* and apoptosis-stimulating protein of p53-2, thereby preserving epithelial polarity and limiting bacterial persistence (62). Additionally, studies in organoids have identified signaling pathways related to cluster of differentiation 44 (CD44), β-catenin, NF-κB activation, and interleukin-8 (IL-8) production as contributors to *H. pylori*–induced gastric epithelial proliferation. Moreover, CRISPR-based screening in gastric organoids further revealed tumor suppressor genes such as *Pten*, *Fbxw7*, and TGF-β pathway components as regulators of gastric tumorigenesis (63). Collectively, these findings demonstrate that gastric organoid systems provide a useful platform for studying host–microbe interactions and their contribution to GC development.

#### 5.1.2 Research on drug resistance mechanisms

Beyond modeling infection-driven gastric carcinogenesis, gastric cancer organoids provide a platform for investigating mechanisms of therapeutic resistance. PDOs preserve patient-specific genomic and phenotypic heterogeneity, enabling functional analysis of therapeutic response.

A key resistance mechanism is dysregulation of DNA damage repair, particularly through Poly (ADP-ribose) polymerase 1 (PARP1) (64). In PDOs, high PARP1 expression was found in organoids sensitive to oxaliplatin (OXA). Inhibition of PARP1 with olaparib, combined with OXA, enhanced antitumor efficacy and overcame acquired resistance, indicating the role of DNA repair pathways in chemoresistance (65).

Other mechanisms of resistance involve membrane-associated signaling and metabolic regulation. Myoferlin (MYOF), a protein involved in vesicle trafficking and receptor signaling, promotes tumor proliferation and resistance by regulating pathways like EGFR signaling (66). In OXA-resistant organoids, MYOF knockdown restored sensitivity to OXA, indicating its role in resistance (67).

Metabolic adaptation also contributes to resistance. The expression of poly(rC)-binding protein 2 (PCBP2) is positively correlated with OXA resistance. Quantitative analysis showed that resistant cancer cells exhibited a 5.39-fold increase in tolerance to OXA compared to their sensitive counterparts (half-maximal inhibitory concentration (IC50): AGSR 33.17 μM vs. AGSWT 6.13 μM). Exogenous lactate supplementation induced dose-dependent upregulation of PCBP2. Notably, PCBP2 knockdown significantly resensitized the models to OXA, which was characterized by a marked reduction in cell viability and a significant increase in apoptosis. These findings suggest an interaction between tumor metabolism, epigenetic regulation, and therapeutic response (68).

Overall, integrative genomic, transcriptomic, and proteomic analyses of PDOs provide insights into resistance pathways, helping identify biomarkers and guiding combination therapies for GC.

### 5.2 Translational prediction: clinical drug sensitivity and personalized oncology

Beyond mechanistic studies of gastric cancer pathogenesis and resistance pathways, PDOs have been increasingly used for functional drug screening and prediction of treatment response. In conventional GC treatment, most patients undergo multimodal therapy centered on surgical resection, yet clinical outcomes remain highly variable (69). With growing recognition of tumor molecular heterogeneity and pharmacogenomic diversity, personalized treatment strategies are increasingly required to improve therapeutic efficacy.

PDOs enable functional evaluation of targeted agents and chemotherapeutic regimens, thereby supporting individualized therapeutic decision-making (70). Because PDOs retain patient-specific genetic and phenotypic characteristics, they may facilitate more precise treatment selection and help reduce unnecessary toxicity.

The clinical predictive value of organoids was demonstrated by Vlachogiannis et al. (8). An organoid biobank derived from patients with metastatic gastrointestinal cancers showed that PDOs recapitulated the histological and genomic features of the original tumors, with a concordance rate of 96%. In functional drug testing, PDOs achieved a negative predictive value of 100% for identifying ineffective therapies and a positive predictive value of 88% for predicting treatment response. These findings suggest that PDOs can assist in identifying ineffective regimens and may support personalized treatment selection. The study also showed that PDO models captured the spatiotemporal evolution of tumors during therapy, providing an experimental platform for monitoring resistance and adjusting treatment strategies.

For example, YOON et al. developed a library of PDOs and conducted *in vitro* chemotherapy drug sensitivity tests. The results demonstrated that organoid sensitivity to fluorouracil, leucovorin, and OXA (FOLFOX) or fluorouracil, leucovorin, OXA, and docetaxel (FLOT) regimens showed a significant positive correlation with postoperative tumor pathological necrosis percentage (Pearson correlation coefficient of 0.87) (71).

Yang et al. further expanded the application of this approach by generating organoids from both primary gastric tumors and lymph node metastases, allowing a comparative analysis of tumor progression and therapeutic response (72).

PDOs also capture substantial heterogeneity between patients in drug sensitivity. In a systematic screening of 41 GC PDO lines, Zhao et al. observed a wide variability in responses to six standard chemotherapeutic agents, reflected by a broad distribution of the area under the values of the dose–response curve (73). In particular, organoids derived from diffuse-type GC exhibited increased sensitivity to 5-fluorouracil and taxane-based therapies, supporting pathologically informed drug selection.

The high biological fidelity of these models is underscored by histological and immunohistochemical analyses, which have confirmed strong concordance in glandular architecture, differentiation status, mucin phenotype, and key marker expression between PDOs and their parent tumors (74). Altogether these observations support that organoids can serve as authentic *in vitro* proxies that preserve the essential biological identity of the original malignancy. Importantly, multiple studies report high agreement between the drug sensitivity profiles of PDOs and clinical treatment outcomes, reinforcing their translational potential (75, 76).

Despite these encouraging findings, several limitations currently restrict the clinical application of PDO models. Current studies are primarily limited by small sample sizes and a significant selective growth bias. For instance, 54% establishment success rate reported by Yoon et al. (71) implies that nearly half of the patient population is excluded from the analysis, particularly those with slow-growing or paucicellular tumors such as signet ring cell carcinoma. This culture-driven selection suggests that a high Pearson correlation coefficient of 0.87 may only represent a metabolically active subset of patients, rather than the highly heterogeneous GC population at large. Consequently, relying on such small, biased cohorts risks overestimating the predictive power of PDOs and limits their generalizability in clinical settings. In addition, current studies adopt heterogeneous response evaluation criteria, including IC50, area under the curve, pathological regression scores, and RECIST-based imaging outcomes, which further complicates cross-study comparison and limits meta-analytic generalization.

Prospective clinical validation is still required to determine whether PDO-guided therapy improves patient outcomes compared with standard treatment. A prospective co-clinical trial by Schmäche et al. addressed this question (77). By establishing a validated area below the curve threshold in an exploratory cohort and confirming its accuracy in an independent validation set, they achieved a 92% accuracy in predicting histological response to neoadjuvant treatment. They proved that results could be delivered within a clinically actionable timeframe of approximately 20 days, circumventing the delays typically associated with organoid expansion.

Further standardization will be necessary to improve cross-center reproducibility. Many current platforms rely on static multi-well culture systems that do not reproduce dynamic pharmacokinetic changes or the complexity of the TME *in vivo*. Incorporating microfluidic systems to simulate drug clearance and establishing standardized, multi-center validated drug sensitivity thresholds may improve reproducibility. Such efforts may facilitate the transition of organoid-based testing from experimental research models toward clinically applicable diagnostic tools (78).

### 5.3 Tumor microenvironment integration: recapitulating the gastric ecosystem

As GC research increasingly considers the tumor niche in addition to tumor-intrinsic mutations, a key limitation of conventional organoid drug testing is the absence of the TME. The TME influences therapeutic response and resistance and consists of stromal and immune cells, soluble factors, extracellular matrix, and vascular and lymphatic components (79). Most organoid cultures rely on Matrigel, a mouse sarcoma–derived extracellular matrix with substantial batch-to-batch variability. Dependence on Matrigel introduces experimental variability and complicates reproducibility and large-scale clinical translation. To better approximate native tissues, the selection of co-culture strategies has become an important aspect of model design (80).

Currently, two main strategies are commonly used: exogenous reconstitution and endogenous preservation (81). The exogenous reconstitution method involves adding external immune cells, such as peripheral blood mononuclear cells or specific CD8^+^ T cells, to pre-established GC organoids (82). This approach enables high-throughput manipulation of individual components.

Using these systems, researchers showed that circular RNA hsa_circ_0136666 promotes GC progression and immune evasion through regulation of the miR-375/protein kinase, DNA-activated, catalytic subunit/programmed death-ligand 1 axis (83). Similarly, human epidermal growth factor receptor 2 activates the phosphoinositide 3-kinase (PI3K)/AKT/mechanistic target of rapamycin pathway, reducing T-cell activity and promoting immune escape (84). However, these additive models often fail to reproduce the native T-cell receptor repertoires and infiltration patterns of the original tumor (85, 86). In contrast, the endogenous preservation approach, typically represented by the ALI culture system, allows GC organoids to be cultured alongside their native stromal cells and tumor-infiltrating lymphocytes. This approach preserves the spatial architecture and immune components of the original tumor tissue (87).

Beyond the choice of platform, the translational utility of GC organoids depends on including specific immune cell subsets that drive the immunosuppressive TME. Recent spatiotemporal multi-omics and single-cell studies suggest that including myeloid-derived suppressor cells and M2-type tumor-associated macrophages is important, as they are primary drivers of the immune-suppressed niche that inhibits T-cell killing capacity (88, 89). Capturing interactions among myeloid cells, epithelial cells, and lymphocytes is therefore important for modeling immune evasion in GC.

Integrated models have already identified key precancerous microenvironmental signaling pathways that promote malignant transformation, with *in vivo* validation that shows that targeting these axes reverses immunosuppression and delays tumorigenesis.

**Figure 4:**
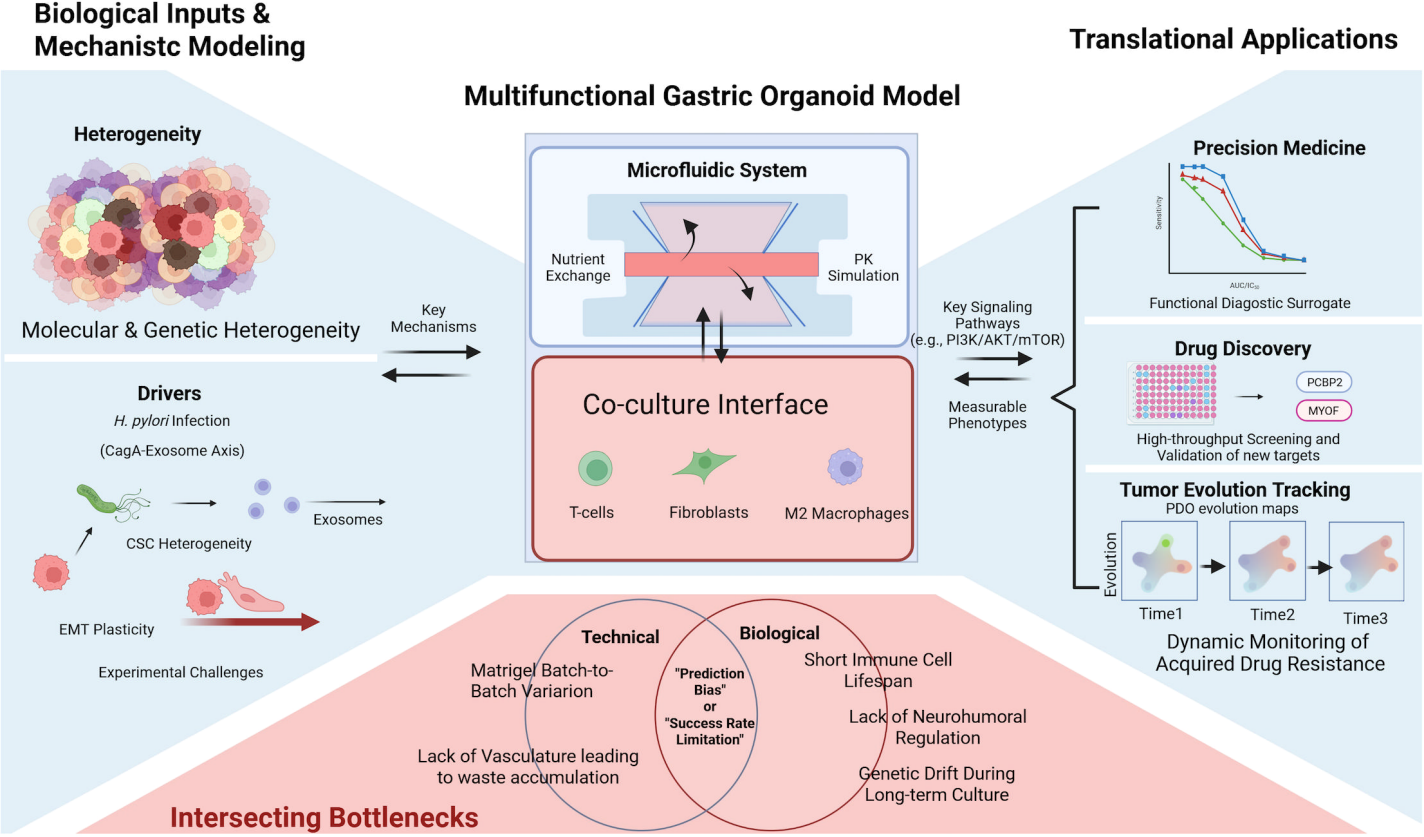
Conceptual framework of gastric cancer organoid applications. This schematic provides a synthetic overview of the role of gastric cancer (GC) organoids in linking basic mechanistic research to clinical translation. (Left) Biological and mechanistic inputs, including genomic heterogeneity, *H. pylori* infection, and tumor microenvironment (TME) factors, serve as the basis for organoid modeling. (Middle) Bioengineered organoid systems provide a platform that links mechanistic studies with translational applications. (Right) Major translational applications include functional precision medicine, high-throughput drug screening, and the tracking of therapy-induced tumor evolution. (Bottom) The red-shaded area highlights the “Intersection of Limitations, “ where biological constraints (e.g., lack of vascularization and neural regulation) and technical challenges (e.g., extracellular matrix variability and high costs) converge. This analytical framework underscores the critical gaps that must be addressed to move GC organoids from bench-side models to standardized clinical tools.

Despite these advances, several technical limitations remain. In current GC models, the absence of blood vessels limits nutrient exchange and promotes the accumulation of acidic metabolic waste. This environment creates metabolic stress that accelerates T-cell dysfunction compared with *in vivo* conditions (90, 91). Such environment-induced dysfunction may lead to an overestimation of drug resistance. Although GC organoid co-culture systems are valuable tools, addressing these limitations through dynamic platforms, such as microfluidic organ-on-a-chip systems that simulate blood flow and maintain nutrient balance, may improve their predictive value for clinical oncology (92–94). These next-generation biomimetic systems may facilitate the study of immuno-oncology mechanisms and support the development of personalized immunotherapy strategies for GC (**Figure 4**).

## 6 Co-culture strategies in gastric organoid models

The increasing recognition of the tumor microenvironment as a critical determinant of GC progression and therapeutic response has driven the development of co-culture strategies to enhance the physiological relevance of organoid models. While conventional organoids preserve epithelial architecture and genetic fidelity, they lack key microenvironmental components, including immune cells, stromal elements, and vascular structures. Co-culture systems therefore represent an essential approach to reconstructing the complex cellular interactions within the gastric tumor ecosystem.

Currently, two principal strategies are employed: exogenous reconstitution and endogenous preservation. The exogenous approach involves the introduction of defined immune or stromal cell populations, such as peripheral blood mononuclear cells or CD8^+^ T cells, enabling controlled and high-throughput investigation of specific cellular interactions. However, these reductionist models often fail to reproduce native immune architecture, including T-cell receptor diversity and spatial infiltration patterns. In contrast, endogenous preservation strategies, particularly ALI systems, retain native stromal components and tumor-infiltrating lymphocytes, thereby preserving tissue architecture and cellular heterogeneity.

Building on these approaches, recent studies (**Tables 2**, **3**) demonstrate a clear transition from simplified binary co-culture systems toward increasingly complex multicellular platforms. Early models primarily focused on direct interactions between tumor organoids and effector immune cells, such as cytotoxic T lymphocytes or natural killer cells, enabling assessment of immune-mediated cytotoxicity. More advanced systems incorporate additional regulatory components, including myeloid-derived suppressor cell–like populations, tumor-associated macrophages, and cancer-associated fibroblasts, thereby enabling simultaneous modeling of anti-tumor immunity and immunosuppressive signaling networks.

**Table 2:** Co-culture models of gastric organoids with immune and stromal components.

Organoids	Co-cultured cells	Co-culture ratio	Culture method	Outcomes	Publication year	Ref
huTGOs (human gastric tumor organoids)	Autologous cytotoxic T lymphocytes (CTLs)	Not reported	Autologous Matrigel-embedded organoid/immune-cell co-culture	HER2 knockdown or inhibition reduces PD-L1 expression in HER2/PD-L1-positive huTGOs, increases CTL proliferation, and enhances organoid cell death.	2021	Chakrabarti J (84)
huTGOs	Autologous CTLs + autologous myeloid-derived suppressor cells (MDSCs) (PMN-MDSC-like suppressor compartment)	Not reported	Autologous Matrigel-embedded organoid/immune-cell co-culture with added MDSCs	MDSCs suppress CTL proliferation and attenuate organoid killing. Cabozantinib reduces myeloid-mediated immunosuppression and improves therapeutic responses in combination settings.	2021	Chakrabarti J (84)
GCOs (gastric cancer organoids)	Peripheral blood mononuclear cells (PBMCs) from healthy donors	50:1 (PBMCs: GCO cells)	Overlay culture method (organoids grown on Matrigel surface rather than fully embedded)	Immune cells freely contact organoids; PBMC-mediated organoid destruction is observed in real time.	2024	Ota, H. (127)
GCOs	Natural killer (NK) cells (isolated from PBMCs, including patient-derived NK cells)	50:1 (NK cells: GCOs)	Overlay culture method	NK cells induce GCO destruction; Concanamycin A reduces this effect; Indicates perforin-dependent killing.	2024	Ota, H. (127)
GC PDOs (gastric cancer patient-derived organoids)	T cells	20:1 (PBMC-derived T cells: GCOs)	PDO immune co-culture system	Demonstrates feasibility of generating patient-specific tumor-reactive T-cell responses; Enables evaluation of tumor-killing effects in GC PDOs.	2024	Zhao Y (73)
GC PDO biobank	Cancer-associated fibroblasts (CAFs)	Not reported	PDO stromal co-culture system	CAF co-culture increases organoid growth and survival; Enhances resistance to 5-FU and oxaliplatin.	2024	Zhao Y (73)
Patient-derived gastric cancer organoids (including poorly cohesive carcinoma, PCC, and non-poorly cohesive carcinoma, NPCC)	CAFs	1:2 (organoids: CAFs)	Direct-contact Matrigel-embedded co-culture system	CAFs form a stromal network surrounding organoids; Enhance organoid proliferation; Induce chemoresistance to multiple chemotherapeutic agents, including 5-FU, oxaliplatin, docetaxel, and irinotecan.	2025	Yin, Y. (128)
Patient-derived gastric cancer assembloids (PDGCAs)	Matched stromal subpopulations (MSC/ECM-like fibroblasts and endothelial cell populations)	1:1–1:4 (optimal ratio 1:4)	Scaffold-free 3D assembloid culture (without Matrigel or Matrigel–fibronectin scaffold)	Stromal integration activates inflammatory and ECM-remodeling programs; Alters drug sensitivity; A 1:4 epithelial-stromal ratio improves structural organization and growth; Increases IL-8 and MMP1 secretion; Enhances tumor–stroma signaling compared with monocultures.	2025	Shapira-Netanelov, I. (129)
Gastric cancer organoids	Stromal cells and immune cells	Not defined (native endogenous stromal and immune composition preserved)	Air–liquid interface (ALI) organoid model	Preserves endogenous epithelial, stromal, and immune components; Maintains native tumor microenvironment architecture compared with epithelial-only PDO cultures.	2024–2025	Polak, R. (10) Kan, L. (81)
Gastric cancer organoids	Immune and stromal components (organ-on-chip/microfluidic systems)	Variable (reconstituted immune–organoid co-culture with adjustable cell ratios)	Microfluidic/organ-on-chip multicellular co-culture models	Enables precise control of microenvironmental conditions (e.g., flow, oxygen, mechanical forces); Supports compartmentalized co-culture; Enables modelling of immune–epithelial interactions and drug responses.	2024–2025	Wang, Q. (104) Papp, D. (122)

huTGOs, human gastric tumor organoids; CTLs, cytotoxic T lymphocytes; PBMCs, peripheral blood mononuclear cells; GCOs, gastric cancer organoids; NK cells, natural killer cells; GC PDOs, gastric cancer patient-derived organoids; PDO, patient-derived organoid; CAFs, cancer-associated fibroblasts; MDSCs, myeloid-derived suppressor cells; PMN-MDSC, polymorphonuclear myeloid-derived suppressor cell; PCC, poorly cohesive carcinoma; NPCC, non-poorly cohesive carcinoma; PDGCAs, patient-derived gastric cancer assembloids; ALI, air-liquid interface; MSC, mesenchymal stem cell; ECM, extracellular matrix; 5-FU, 5-fluorouracil; HER2, human epidermal growth factor receptor 2; PD-L1, programmed death-ligand 1.

**Table 3:** Co-culture models of gastric organoids with diverse cell types and microorganisms in recent studies.

Organoids	Co-cultured cells	Co-culture ratio	Culture method	Outcomes	Publication year	Ref
mFGOs (mouse fundic gastric organoids)	Immortalized stomach mesenchymal cells (ISMCs)	Not reported	Transwell-based epithelial–mesenchymal co-culture (Matrigel + Transwell system)	Recapitulates mature fundic epithelial cell types; Supports studies of gastric physiology; Regeneration; Host–pathogen interactions.	2016	Bertaux-Skeirik, N. (130)
PSC-derived human gastric organoids (antral)	Intrinsic mesenchymal compartment	N/A (intrinsic co-development)	PSC-directed differentiation + 3D Matrigel culture	Generated epithelial and mesenchymal components; Provided an early multicellular gastric organoid platform.	2014	McCracken, K. W. (16)
Human and murine gastric organoids (adult stem cell-derived)	*Helicobacter pylori*	Not specified	3D Matrigel culture; bacteria delivered by luminal microinjection	Established gastric organoid infection model for host–pathogen interaction studies.	2015	Bartfeld, S. (102)
Human gastric organoid-derived primary epithelial monolayers	*Helicobacter pylori*	(multiplicity of infection)= 100	3D gastric spheroids/organoids generated in Matrigel, followed by transfer to collagen-coated 2D monolayer culture for infection	Recapitulated key hallmarks of *H. pylori* infection; CagA phosphorylation; Hummingbird phenotype; Inflammatory responses; No microinjection required.	2016	Schlaermann, P. (28)
Human gastric mucosoid cultures	*H. pylori*	(multiplicity of infection)= 100	Air–liquid interface (ALI)/Transwell mucosoid co-culture	Modeled polarized mucus barrier; Enabled long-term apical infection; Recapitulated mucosal defense mechanisms.	2019	Boccellato, F. (21)
Human gastric organoids	*H. pylori* + monocyte-derived dendritic cells (MoDCs)	~5 × 10^5^ MoDCs per ~80 spheroids (not standardized per organoid)	Microinjection of *H. pylori* into the organoid lumen, followed by addition of moDCs to the basolateral compartment (Transwell-like setup)	MoDCs migrate to the basolateral epithelium; Extend dendrites; Phagocytose luminal bacteria; Epithelial chemokine secretion (CXCL1, CXCL16, CXCL17, CCL20) mediates DC recruitment; *H. pylori* infection enhances DC recruitment.	2019	Sebrell, T. A. (131)
Human gastric organoids	*Helicobacter pylori* + autologous immune cells (dendritic cells and cytotoxic T lymphocytes, CTLs)	Not explicitly reported (estimated ~10^3^–10^4^ cells/organoid)	Microinjection of *H. pylori* into organoid lumen followed by co-culture with immune cells	*H. pylori* induces epithelial PD-L1 expression via Hedgehog signaling, leading to suppression of CTL activity; Contributes to immune evasion.	2019	Holokai, L. (132)
Mouse primary gastric epithelial organoid-derived monolayers	*H. pylori* + bone marrow–derived macrophages (WT or Nod1−/−)	MOI = 30 (bacteria: epithelial cells)	Transwell co-culture of infected epithelial monolayers with macrophages	Nod1 suppresses pro-inflammatory cytokine production; Regulates macrophage polarization during *H. pylori* infection.	2019	Suarez, G. (133)
Human gastric organoids	*H. pylori*	MOI 50	Human organoid infection model	CagA–ASPP2 interaction disrupts epithelial cell polarity and promotes bacterial colonization.	2020	Buti, L. (62)
Human gastric organoids (GOFlowChip)	Monocyte-derived dendritic cells (MoDCs)	Not explicitly specified	Microfluidic tissue chip (GOFlowChip) with dynamic flow; VitroGel^®^ ORGANOID-3 hydrogel-based 3D co-culture	Enhanced dendritic cell chemotaxis; Migration; Immune cell–epithelial interactions compared to Matrigel.	2021	Cherne, M. D. (27)
Human gastric antral organoid-derived epithelial cells	Primary gastric mesenchymal stromal cells (gMSCs)	4:1 (epithelial:gMSC; ~5 × 10^6^ epithelial cells mL^–1^ vs ~1.25 × 10⁶ gMSCs mL⁻¹)	Organoid-based human stomach microphysiological system (hsMPS); hAO-derived epithelial cells seeded in the luminal channel and gMSCs in the abluminal channel of a two-channel PDMS chip separated by a porous PET membrane; continuous medium flow (~60 μL h^–1^)	Enhanced epithelial–mesenchymal interactions; Sustained gastric progenitor activity; Promoted mesh-like mucus barrier formation; Increased MUC5AC, MUC6, TFF1, and TFF2; Improved epithelial junctional integrity; Enabled modeling of *H. pylori* mucosal defense.	2023	Jeong, H. J. (121)
Human gastric organoid-derived epithelial cells	*Helicobacter pylori*	Not specified	Organoid-on-a-chip (Transgel) system: organoid-derived gastric epithelial cells seeded on a geometrically patterned hydrogel within a PDMS device with apical–basal bilateral access; apical acidic or neutral conditions; long-term infection under dynamic medium exchange	Supports niche formation; promotes bacterial microcolony formation at cell junctions; reveals cell type–specific responses; progenitor and immature cells exhibit cytokine responses; mature pit cells upregulate junctional and antimicrobial genes (e.g., DUOX2/DUOXA2) under acidic conditions.	2025	Hofer, M. (120)
Patient-derived precancerous gastric organoids/cell models	Fibroblasts	Not reported	Co-culture (organoid–fibroblast)	NAMPT signaling activates fibroblasts via ITGA5/ITGB1; Promotes precancerous progression-related programs.	2025	Gao, P. (88)
Patient-derived precancerous gastric organoids/cell models	Macrophages (M1/M2)	Not specified	Transwell co-culture (macrophage–epithelial/organoid)	Macrophage-derived AREG activates EGFR/ERBB2 signaling; promotes proliferation; induces PD-L1-associated malignant transition programs.	2025	Gao, P. (88)
Patient-derived gastric organoids (NGOs/TGOs)	*Helicobacter pylori* + Epstein-Barr virus (EBV)	Not explicitly stated (EBV MOI ≈ 10; *H. pylori* concentration normalized by OD (no fixed MOI)	Microinjection into organoid lumen	Induced structural alterations (folding, curling); Enhanced cell proliferation and morphogenesis; Upregulated TFF1, VIL1, and Lgr5; Promoted internalization of *H. pylori*; Activated EBV lytic replication.	2025	Liu, L. (134)

mFGOs, mouse fundic gastric organoids; ISMCs, immortalized stomach mesenchymal cells; PSC, pluripotent stem cell; *H. pylori, Helicobacter pylori*; ALI, air–liquid interface; MoDCs, monocyte-derived dendritic cells; CTLs, cytotoxic T lymphocytes; MOI, multiplicity of infection; WT, wild type; NOD1, nucleotide-binding oligomerization domain-containing protein 1; GOFlowChip, gastric organoid flow chip platform; gMSCs, gastric mesenchymal stromal cells; hsMPS, human stomach microphysiological system; hAO, human antral organoid; PDMS, polydimethylsiloxane; PET, polyethylene terephthalate; μL, microliter; MUC5AC, mucin 5AC; MUC6, mucin 6; TFF1, trefoil factor 1; TFF2, trefoil factor 2; DUOX2, dual oxidase 2; DUOXA2, dual oxidase maturation factor 2; NAMPT, nicotinamide phosphoribosyltransferase; ITGA5, integrin subunit alpha 5; ITGB1, integrin subunit beta 1; M1/M2, macrophage polarization states; AREG, amphiregulin; EGFR, epidermal growth factor receptor; ERBB2, erb-b2 receptor tyrosine kinase 2; PD-L1, programmed death-ligand 1; NGOs, normal gastric organoids; TGOs, tumor gastric organoids; EBV, Epstein–Barr virus; OD, optical density.

Functionally, immune-integrated organoid models recapitulate key aspects of tumor–immune interactions, including both cytotoxic responses and immune suppression. Co-culture with CTLs, PBMCs, and NK cells induces tumor cell killing, whereas the presence of suppressive myeloid populations attenuates T-cell proliferation and cytotoxicity. In parallel, stromal co-culture systems highlight the critical role of non-immune components, with cancer-associated fibroblasts promoting tumor growth, survival, and resistance to therapy. Advanced assembloid models incorporating fibroblasts and endothelial cells further enhance tissue organization and enable investigation of extracellular matrix remodeling and angiogenic signaling.

Beyond tumor–immune–stroma interactions, gastric organoid co-culture models have been extended to host–pathogen systems. Co-culture with *Helicobacter pylori* or Epstein–Barr virus enables the study of infection-driven gastric carcinogenesis, including epithelial remodeling, inflammatory signaling activation, and immune modulation. These models recapitulate key early events in gastric tumorigenesis within a controlled three-dimensional environment.

From a methodological perspective, these advances are accompanied by increasing diversification of culture platforms. In addition to conventional Matrigel-based systems, alternative configurations such as ALI cultures, Transwell systems, scaffold-free assembloids, and microfluidic organ-on-chip technologies have been developed to improve spatial organization, cellular accessibility, and environmental control. In particular, microfluidic systems enable dynamic perfusion and more physiologically relevant modeling of nutrient exchange, drug exposure, and immune cell trafficking.

## 7 Challenges

Since Clevers et al. established a 3D culture system for intestinal stem cells, organoid technology has achieved groundbreaking progress in GC research. Despite these advances, GC organoid co-culture systems remain at a translational bottleneck. Moving organoid systems toward routine clinical use requires addressing biological, engineering, and regulatory barriers. While several technical limitations of gastric cancer organoid models have been discussed in previous sections, this section focuses on broader translational challenges including model standardization, clinical validation, and future technological integration.

### 7.1 Organoids as dynamic models of tumor evolution

#### 7.1.1 Tumor plasticity and epithelial–mesenchymal transition

In cancer research, cell plasticity is defined as the ability of cells to be reprogrammed toward a different fate and change their identity in response to intrinsic or extrinsic factors (95). A key clinical manifestation of this plasticity is epithelial-mesenchymal transition (EMT), which is now recognized not as a binary process, but as a fluid transition through a series of intermediate states known as epithelial-mesenchymal plasticity (96). Organoids serve as a pivotal platform to recapitulate these non-genetic dynamic evolutions induced by environmental signals (95). However, because most organoid cultures lack stromal, immune, and vascular components, the full spectrum of EMT-associated plasticity and microenvironment-driven state transitions may not be completely reproduced *in vitro.*

#### 7.1.2 Cancer stem cell dynamics and niche-dependent selection

Beyond EMT-associated plasticity, tumor evolution is also shaped by the dynamic hierarchy of cancer stem cells (CSCs) and their interaction with the surrounding niche. The maintenance and hierarchy of CSCs are fundamentally governed by their interaction with the surrounding niche. Landmark studies have demonstrated that single Lgr5+ stem cells possess remarkable self-organizing capabilities, enabling them to build complex crypt-villus structures *in vitro* even in the absence of a non-epithelial mesenchymal niche (12).

However, this hierarchy is not a unidirectional process; rather, it reflects a high degree of niche-dependent plasticity. Non-stem cells can re-acquire stemness properties when stimulated by specific microenvironmental signals, such as Wnt or Notch signaling, highlighting a dynamic state of selection and competition (97). Organoid culture systems serve as a critical platform to dissect these dynamics, revealing how specific culture conditions can selectively enrich certain CSC subpopulations, thereby driving the long-term clonal evolution and heterogeneity of the tumor.

Collectively, although organoid cultures preserve key stemness hierarchies and enable dynamic state transitions, the artificial niche composition and selective growth conditions may unintentionally bias CSC representation, potentially reshaping clonal dominance and altering the evolutionary trajectory compared with the *in vivo* tumor ecosystem.

#### 7.1.3 Clonal selection, culture-induced genetic drift, and implications for therapy modeling

In addition to stem-cell–driven hierarchy, tumor evolution is further shaped by clonal selection and genetic drift during long-term organoid culture. While organoids are generally recognized for maintaining genomic stability over long-term culture, they are not immune to the dynamics of clonal selection and genetic drift during *in vitro* expansion. Research has shown that human intestinal stem cells can accumulate a low number of spontaneous mutations during prolonged culture, though they remain phenotypically stable under homeostatic conditions (98).

Importantly, serial sampling of patient-derived organoids during treatment provides a unique opportunity to model therapy-induced evolutionary trajectories, enabling the identification of resistant clones emerging under pharmacological pressure.

However, modeling tumor evolution and therapeutic selection in PDO systems faces several biological and technical hurdles (80, 99, 100). During prolonged passaging, organoids may suffer from genomic drift or lose differentiation capacity, potentially causing drug responses to deviate from the original tumor (101, 102). These limitations complicate the interpretation of therapy-response modeling and long-term evolutionary dynamics.

To address both microenvironmental instability and long-term culture limitations, several engineering strategies have been explored. Cytokine supplementation (IL-2 and IL-15), stromal support cells, and microfluidic perfusion can partially stabilize immune populations and improve culture longevity (103). Furthermore, organ-on-chip platforms still face challenges in modeling stable luminal acidic pH gradients and mucus turnover. Although dual-channel designs promote epithelial maturation, real-time monitoring of gastric mechanical cues and drug penetration is still limited (104–106).

To mitigate the risks of genomic drift associated with such complex long-term systems, routine genomic validation via low-pass whole-genome sequencing should be implemented every 5 to 10 passages, establishing a clear biological expiration threshold for each line (103, 107).

### 7.2 Engineering and standardization challenges

Beyond tissue quality, the field faces ongoing debates about standardization and methodology. In particular, the optimal extracellular matrix composition, the degree of culture medium standardization, and the long-term impact of passaging on genomic stability remain actively discussed topics within the organoid research community. These unresolved methodological variations can significantly influence organoid growth dynamics, cellular composition, and drug-response profiles, thereby complicating cross-study comparisons and limiting the reproducibility of experimental findings.

A major hurdle in standardization is the inherent variability of recombinant proteins, which often suffer from batch-to-batch instability. Emerging strategies advocate for the transition toward chemically defined media by replacing expensive and unstable growth factors with small-molecule mimetics (57). This transition is crucial for reducing cost and ensuring that pharmacokinetic and drug-sensitivity assays are performed under chemically precise conditions.

Furthermore, beyond issues of media composition and standardization, current gastric organoid systems also face limitations in long-term functional maintenance. Under existing culture conditions, maintaining functional parietal cell activity and stable epithelial polarization beyond three weeks remains challenging (108–110). Recent studies suggest that optimized extracellular matrices combined with microfluidic perfusion may help preserve gastric gland architecture and sustain acid-secreting cell populations (32, 111).

In parallel, improving long-term immune–organoid co-culture stability will require integrated niche engineering strategies, including stromal cell incorporation, cytokine supplementation, and vascularized microfluidic systems that better replicate nutrient exchange and immune trafficking.

### 7.3 Clinical validation and regulatory considerations

The choice of organoid culture mode should be guided by the intended biological or clinical application rather than by a single universally superior platform, highlighting the need for context-specific standardization in future translational studies.

In advanced GC, clinical decisions often require rapid intervention, whereas the establishment and drug testing of PDOs can exceed the optimal therapeutic window. In addition, variable organoid establishment efficiency may introduce patient-selection bias and limit the generalizability of PDO-guided treatment strategies. These limitations highlight the need for optimized culture protocols and improved bioengineering approaches to broaden the clinical applicability of organoid-based precision oncology.

From a clinical perspective, a critical bottleneck is the lack of statistically powered validation. While the exact cohort size required for adequately powered prospective validation remains to be defined, existing PDO studies with sample sizes ranging from approximately 50 to 160 patients suggest that substantially larger patient cohorts will be required to achieve statistically reliable survival analyses and predictive validation, particularly given the high inter-patient heterogeneity of GC (73, 112, 113).

Finally, the broader clinical implementation of PDOs also depends on regulatory and economic considerations. High production costs and the absence of insurance coverage currently restrict PDO use to specialized research centers. Demonstrating the cost-effectiveness of organoid-guided therapy will be important for broader adoption. For example, this approach may reduce the use of ineffective or toxic treatments (114). Furthermore, the field requires standardized protocols for quality control and clearer government regulations. PDO platforms must navigate complex regulatory frameworks, such as the *In Vitro* Diagnostic Regulation (IVDR), before they can be fully integrated into standard hospital workflows (115).

### 7.4 Ethical considerations in patient-derived organoid research

The clinical translation of patient-derived organoid (PDO) technology raises several important ethical issues, particularly regarding informed consent, genomic data privacy, and ownership of patient-derived biological materials. The informed consent process must be redefined to cover the immortal nature of organoid lines and potential future commercial applications. Data privacy remains a concern, as high-fidelity genomic data carries a risk of donor re-identification, necessitating stringent de-identification protocols (116, 117). Researchers must also address sample ownership using a custodianship model, treating living tumor tissues as trust property of the patients to prevent financial gain from raw tissue while allowing model patenting (118, 119). By establishing transparent standard operating procedures and following the International Society for Stem Cell Research, the scientific community can maintain public trust and move PDO-based diagnostics into routine precision medicine (120–122). Despite these regulatory, ethical, and technical challenges, emerging interdisciplinary technologies are opening new avenues for the next generation of organoid platforms.

## 8 Future perspectives

As gastric cancer organoid technology transitions into its next generation, the focus is shifting from simple 3D cultures toward systems-level integration. This integrative approach may help address current limitations, including the loss of spatial architecture and the limited scalability of drug screening.

Central to this evolution is the integration of multi-omics data. Platforms like StomachDB synthesize genomics, transcriptomics, and proteomics to bridge the gap between genotype and phenotype. Instead of mere descriptive observations, this integration provides a holistic understanding of tumor evolution and helps identify specific therapeutic targets across multiple molecular layers (123).

To address the loss of spatial context in 3D cultures, spatial transcriptomics is becoming indispensable. This technology maps the spatial organization of the TME, such as tumor-infiltrating lymphocytes and stromal cells. Capturing these spatial relationships is critical, as they are strongly correlated with patient prognosis and treatment resistance (124).

In parallel, AI-based predictive modelling is reshaping precision oncology by enabling the scalable analysis of high-dimensional datasets, including imaging, transcriptomics, and pharmacological readouts. Advanced techniques such as deep learning and transfer learning enhance phenotypic profiling, drug sensitivity prediction, and patient stratification (125). Moreover, the synergy between AI and organoid-on-chip systems allows for real-time feedback and dynamic modeling of organoid development, addressing the inherent variability of current culture techniques (126). These next-generation approaches may facilitate the transition of tumor organoids from structural models to more standardized platforms with functional readouts for translational assessment (78).

Looking forward, the next five years are expected to focus on establishing standardized organoid biobanks and multi-center validation trials. Within the next decade, integration of organoid-guided drug screening with AI-assisted predictive modeling may enable routine clinical decision support for precision oncology. Together, these advances are expected to facilitate the transition of tumor organoids from structural models to standardized functional platforms linking laboratory research with clinical applications.

## Glossary

GC: Gastric cancer
3D: three-dimensional
*H. pylori*: *Helicobacter pylori;* PDOs, patient-derived organoids
TME: tumor microenvironment
EGF: epidermal growth factor
iPSC: induced pluripotent stem cell
ALI: air-liquid interface
2D: two-dimensional
PDX: patient-derived xenograft
ASC: adult stem cell
CagA: cytotoxin-associated gene A
FOLFOX: fluorouracil, leucovorin, and oxaliplatin
FLOT: fluorouracil, leucovorin, oxaliplatin, and docetaxel
PI3K: phosphoinositide 3-kinase
AKT: protein kinase B
PARP1: poly(ADP-ribose) polymerase 1
MYOF: myoferlin
EGFR: epidermal growth factor receptor
*PCBP2*: *poly(rC)-binding protein 2*
EMT: epithelial-mesenchymal transition
CSCs: cancer stem cells
IC50: half-maximal inhibitory concentration
CRISPR: clustered regularly interspaced short palindromic repeats
PSC: pluripotent stem cell
OXA: oxaliplatin
BMP: bone morphogenetic protein
FGF: fibroblast growth factor
LGR5: leucine-rich repeat–containing G protein-coupled receptor 5
SOX: S-1 plus oxaliplatin
IL-8: interleukin-8
CD44: cluster of differentiation 44.
2D: 2-dimensional
PDO: Patient-derived organoid
PDX: Patient-derived tumor xenograft
ALI: Air-Liquid Interface
